# Characterization of a novel cell penetrating peptide derived from human Oct4

**DOI:** 10.1186/2045-9769-3-2

**Published:** 2014-01-31

**Authors:** Eva Harreither, Hanna A Rydberg, Helene L Åmand, Vaibhav Jadhav, Lukas Fliedl, Christina Benda, Miguel A Esteban, Duanqing Pei, Nicole Borth, Regina Grillari-Voglauer, Oliver Hommerding, Frank Edenhofer, Bengt Nordén, Johannes Grillari

**Affiliations:** 1 Department of Biotechnology, University of Natural Resources and Life Sciences, Muthgasse 18, 1190 Vienna, Austria; 2 Department of Chemical and Biological Engineering/Physical Chemistry, Chalmers University of Technology, Kemivägen 10, S-412 96 Gothenburg, Sweden; 3 Evercyte GmbH, Muthgasse 18, 1190 Vienna, Austria; 4 ACIB GmbH, Austrian Center of Industrial Biotechnology, Petersgasse 14, 8010 Graz, Austria; 5 Key Laboratory of Regenerative Biology, Chinese Academy of Sciences, and Guangdong Provincial Key Laboratory of Stem Cells and Regenerative Medicine, South China Institute for Stem Cell Biology and Regenerative Medicine, Guangzhou Institutes of Biomedicine and Health, 510530 Guangzhou, China; 6 Stem Cell Engineering Group, Institute of Reconstructive Neurobiology, University of Bonn - Life & Brain Center and Hertie Foundation, Sigmund-Freud Strasse 25, D-53105 Bonn, Germany; 7 Stem Cell and Regenerative Medicine Group, Institute of Anatomy and Cell Biology, Julius-Maximilians-University Würzburg, Koellikerstrasse 6, D-97070 Würzburg, Germany; 8 Christian Doppler Laboratory on Biotechnology of Skin Aging, University of Natural Resources and Life Sciences, Muthgasse 18, 1190 Vienna, Austria

**Keywords:** Cell penetrating peptides, Oct4, Penetratin, Homeodomain transcription factors, Cellular internalization, Reprogramming

## Abstract

**Background:**

Oct4 is a transcription factor that plays a major role for the preservation of the pluripotent state in embryonic stem cells as well as for efficient reprogramming of somatic cells to induced pluripotent stem cells (iPSC) or other progenitors. Protein-based reprogramming methods mainly rely on the addition of a fused cell penetrating peptide. This study describes that Oct4 inherently carries a protein transduction domain, which can translocate into human and mouse cells.

**Results:**

A 16 amino acid peptide representing the third helix of the human Oct4 homeodomain, referred to as Oct4 protein transduction domain (Oct4-PTD), can internalize in mammalian cells upon conjugation to a fluorescence moiety thereby acting as a cell penetrating peptide (CPP). The cellular distribution of Oct4-PTD shows diffuse cytosolic and nuclear staining, whereas penetratin is strictly localized to a punctuate pattern in the cytoplasm. By using a Cre/loxP-based reporter system, we show that this peptide also drives translocation of a functionally active Oct4-PTD-Cre-fusion protein. We further provide evidence for translocation of full length Oct4 into human and mouse cell lines without the addition of any kind of cationic fusion tag. Finally, physico-chemical properties of the novel CPP are characterized, showing that in contrast to penetratin a helical structure of Oct4-PTD is only observed if the FITC label is present on the N-terminus of the peptide.

**Conclusions:**

Oct4 is a key transcription factor in stem cell research and cellular reprogramming. Since it has been shown that recombinant Oct4 fused to a cationic fusion tag can drive generation of iPSCs, our finding might contribute to further development of protein-based methods to generate iPSCs.

Moreover, our data support the idea that transcription factors might be part of an alternative paracrine signalling pathway, where the proteins are transferred to neighbouring cells thereby actively changing the behaviour of the recipient cell.

**Electronic supplementary material:**

The online version of this article (doi: 10.1186/2045-9769-3-2) contains supplementary material, which is available to authorized users.

## Introduction

Cell penetrating peptides (CPPs) - also known as protein transduction domains (PTDs) - are an intensively studied, yet diverse class of peptides in regard to amino acid composition, size, charge and structure. Their joint feature is the mediation of the internalization of a peptide or a protein and, in most cases, a conjugated cargo. Classifications are either based on the physico-chemical nature of the sequence (primary-, secondary-, or non-amphiphatic) [1] or their origin (protein-derived, chimeric or synthetic peptides) [2].

The first CPPs, Tat and penetratin, were described already in the late 1980s and early 1990s, and are still among the most studied CPPs today [3]. Tat is derived from amino acids 48-60 of HIV-1 trans-activator of the transcription (Tat) protein [4, 5], while penetratin derives from the *Drosophila melanogaster* homeodomain protein Antennapedia (Antp). Antp is a transcription factor and its 60 aa homeodomain (pAntp) can be unconventionally secreted without the need of a signal peptide [6]. Consequently, it can be uptaken by neighbouring cells in a receptor-independent way [7]. Penetratin, a 16 amino acid peptide, corresponds to the third helix of the Antp homeodomain and has been shown to be sufficient for uptake of the whole protein [8]. In previous studies it was shown that internalization of penetratin relies mainly on endocytosis [9], but direct translocation has also been proposed [10–12]. Still, the mechanism of uptake remains debatable [3].

Detailed characterization of the mechanisms guiding peptide internalization is desired, and i.e. the interaction of penetratin and its derivatives with cellular membrans has been intesively studied using lipid model systems [13]. These studies have clarified that upon binding of penetratin to anionic lipid membranes, the peptide changes its secondary structure and adopts either an α-helical or β-sheet shaped structure, depending on the peptide/lipid ratio [14, 15].

Ever since the discovery of penetratin, a number of other peptides as well as proteins from the large family of homeodomain transcriptional regulators have been assessed for their capacity to cross cellular membranes. Prominent examples of internalized whole proteins or the homeodomain helix in combination with cargo molecules are Hoxa5, Hoxc8, PDX-1 or Engrailed-2 [16–19]. However, not all homeodomain peptides or proteins are efficiently taken up by cells [20]. In contrast, uptake of Pax-4, a paired-box transcription factor also containing a homeodomain, has been shown to depend upon the paired domain rather than the homeodomain [21].

One homeodomain protein that previously has not been tested for containing a functional PTD is human Oct4. Oct4 is a prominent member of the POU-family of transcription factors containing two distinct DNA-binding domains, the POU-specific domain and the homeodomain [22]. It is necessary for maintaining the pluripotent state of embryonic stem cells [23], but has probably raised most interest as a key factor for cellular reprogramming of somatic cells into induced pluripotent stem cells (iPS) [24].

While several techniques to non-integratively transfect cells with reprogramming factors like mRNA or CPP-tagged proteins [25–29] have been reported, a simple and safe approach adding no genetically altered authentic recombinant protein has not been tested so far.

Therefore, we explored if the third helix of the human Oct4 homeodomain (Oct4-PTD) might in principle be able to translocate into living cells and mediate cargo uptake. We compared this activity as well as its secondary structure, membrane interaction and cytotoxicity to the well characterized CPP penetratin.

We report that Oct4-PTD-mediated uptake is very efficient, and that already 1 hour after exposure, FITC-labelled Oct4-PTD localizes to the nucleus and diffusely to the cytoplasm. Structural analysis of the peptides using circular dichroism (CD) showed that upon binding to large unilamellar vesicles (LUVs), a helical structure of Oct4-PTD is only observed if the FITC label was present on the N-terminus of the peptide. In contrast, penetratin shows a helical structure independently of the fluorescence moiety.

Moreover, we show that recombinantly expressed and purified Oct4-PTD-Cre fusion protein successfully translocates into reporter cells when added to the medium and recombines loxP-modified marker genes. Finally, a weak but significant uptake of recombinantly expressed native human Oct4 was observed. We therefore suggest that uptake of Oct4 might be a further contribution towards establishing safe methods to generate iPSCs.

## Material and methods

### Materials

Peptides (>95% purity) were purchased from Biomatik (Cambridge, Ontario, Canada). For labelled peptides the N-terminal fluorescein isothiocyanate (FITC) label was conjugated by an Acp (amino caproic acid) linker. The peptides were delivered as lyophilized acetate salts, dissolved in MQ-water and stored at -20°C as aliquots. Cell culture media were from PAA Laboratories and Biochrom AG (Berlin, Germany), heparin sodium was purchased from Serva (Heidelberg, Germany) and buffer salts were from Sigma. FM 4-64 dye and 7-aminoactinomycin D (7-AAD) were from Invitrogen. 1-Palmitoyl-2-oleoyl-sn-glycero-3-phosphocholine (POPC), 1-Palimitoyl-2-oleoyl-sn-glycero-3-phosphoglycerol (POPG) were from Larodan Fine Chemicals (Malmö, Sweden).

### Cell culture

Chinese Hamster Ovary cells (CHO-K1) were cultured in Ham’s F12 medium supplemented with 10% fetal calf serum and 2 mM L-Glutamine. RPTEC/TERT1 cells [30] were cultivated in ProxUp medium (Evercyte GmbH, Austria). CV1-5B cells and BJ foreskin fibrobrasts were cultured in DMEM supplemented with 10% FCS, 1× NEAA, 1× Sod.-Pyruvat (all Life technologies, Carlsbad, CA). Cell lines were maintained under antibiotic free conditions at 37°C and 5% CO2.

### Flow cytometry

For quantitative analysis of uptake 10,000 CHO-K1 cells were seeded per well of a 96-well plate 48 h prior to the experiment and cultured to approximately 80% confluence. The cells were washed with serum-free (SF) Ham’s F12 and subsequently incubated with indicated concentrations of FITC-labelled peptides diluted in SF-medium for 60 min at 37°C, 5% CO_2_.

Following incubation cells were washed twice with 20 mM HEPES/150 mM NaCl, pH 7.4, supplemented with 100 μg/ml Heparin in the first wash step to remove membrane bound peptide from the cell surface [31]. Cells were detached from the wells and uptake was immediately analyzed using a Guava EasyCyte 8HT flow cytometer (Millipore). For every concentration step, 4 individual wells were seeded and analyzed (n = 4) and experiments were repeated at 3-4 separate occasions (N = 3-4). Untreated cells were used to set the gate on live cells (FSC/SSC) and fluorescence emission of 5000 cells was recorded through a 525/30 nm filter. Uptake data are reported as mean fluorescence intensity (MFI) of all gated cells in each sample. For statistical analysis of significance Student’s t-tests were performed.

### Cell proliferation using MTT

The MTT assay is based on the detection of the reduction of yellow MTT (3-(4,5-Dimethylthiazol-2-yl)-2,5-diphenyltetrazolium bromide) to purple formazan which can be detected photometrically at 530 nm [32]. RPTEC/TERT1 cells were seeded in 96-well plates and cultured to 90% confluence. Cells were incubated in triplicates with 0; 1.25; 2.5; 5.0;10.0; 20.0; 40.0; and 80 μM of unlabelled peptide and incubated for 1 h or 24 h in serum free medium at 37°C, 5% CO_2_. The mean value of each concentration point was compared with the negative control. Endpoints of the measurements were plotted and a 1-way ANOVA was calculated using Friedmann Test in GraphPad Prism.

### Haemolysis

For testing the haemolytic activity of both labelled and unlabelled peptides, fresh human red blood cells (RBCs) were isolated from whole blood using a Ficoll gradient. The cells were washed twice in PBS and were subsequently resuspended in PBS to gain a final concentration of 4% (v/v). 100 μl of RBCs were substituted with 100 μl of the respective peptide solution diluted in PBS to gain a final peptide concentration of 5, 15 or 45 μM, followed by incubation at 37°C for 1 h. As negative control, RBCs were incubated in PBS and as positive control, corresponding to 100% hemolysis, cells were incubated with 0.05% Triton-X100 in PBS. Cells, treated with FITC-labelled peptides were blanked against a dilution of respective peptide in PBS and an absorbance scan of the FITC spectrum (in PBS) confirmed no peak at 405 nm. All samples were analyzed in three biological replicates. Absorbance was detected at 405 nm on a Tecan Infinite 200 PRO Microplate Reader and hemolytic activity was calculated following [33].


### Confocal imaging

For confocal live cell imaging CHO-K1 cells were seeded at a density of ~14,000 cells/cm^2^ in round glass bottomed dishes 48 h prior to microscopy. For imaging, cells were rinsed with SF-medium and incubated with FITC-labelled peptides at indicated time points (15, 30 60 min) and concentrations (5 or 10 μM) in the presence or absence of 5 μg/ml FM 4-64 dye at 37°C or 4°C. Microscopy was performed on a Leica TCS SP confocal system equipped with a HCX PL APO CS 63× objective. For excitation of FITC the 488 nm laser line of an Ar laser was used and the 543 nm laser of a He/Ne laser was used for excitation of FM 4-64. Sequential scanning mode was used to avoid bleed through between the channels; laser and photomultiplier settings were kept constant within each experiment. Image optimization of contrast, gain and brightness was performed to the same extent within each experiment and intensities of the respective panels are therefore comparable.

### Preparation of LUVs

Large unilamellar vesicles (LUVs) were prepared by extrusion as previously described [34]. In brief, a solution of POPC/POPG (80/20 molar ratio) dissolved in chloroform was mixed in a round bottom flask, after which the solvent was completely evaporated. Vesicles were formed by dispersion of the lipid film in 10 mM NaPO_4_ buffer, pH 7.4 under vortexing (5 min), followed by five freeze-thaw cycles (liquid nitrogen/50°C) and extruding 21 times through Nucleopore polycarbonate filters with a pore diameter of 100 nm using a LiposoFast-Pneumatic extruder (Avestin, Canada). LUVs used for LD studies were prepared in the same phosphate buffer described above containing 50% (w/w) sucrose.

### Circular dichroism (CD) spectroscopy

Circular dichroism (CD) spectroscopy was performed using a Chirascan instrument (Applied Photophysics, Leatherhead, UK). The instrument was flushed with nitrogen at a flow rate of 5 l/min. Far-UV CD spectra were recorded in the region between 185 nm and 270 nm, the path length was 2 mm, 1 nm steps were performed at a bandwidth of 2 nm and the scan time per point was set to 0.5 s. 20 repeats were averaged for each sample and baseline-correction was performed by subtracting appropriate blanks. The experiments were repeated at three different occasions. 4 μM of peptide solution was measured in the absence or presence of 0.4 mM LUVs (peptide:lipid-ratio 1:100) in 10 mM NaPO_4_ buffer, pH 7.4. Since the signal of unlabelled Oct4-PTD in this concentration was very week, an additional measurement with 12 μM unlabelled Oct4-PTD was performed, with a peptide to lipid ration of 1:33.

### Linear dichroism (LD) spectroscopy

Linear dichroism (LD) is the difference in absorption between linearly polarized light parallel (A_||_) and perpendicular A_⊥_ to an orientation axis [35].


LD spectra were recorded on a Chirasacan spectropolarimeter (Applied Photophysics, Leatherhead, UK) equipped with a Couette flow cell. The flow cell consists of two cylinders and the applied sample was aligned in the gap between them resulting in an optical path length of 1 mm. Spectra were measured between 185 nm and 600 nm at a shear flow of 3100 s^-1^. The spectra were baseline corrected by subtracting the corresponding spectrum without rotation [36]. 14 μM of unlabelled peptide was added to 1.4 mM LUVs in a 10 mM phosphate buffer (pH 7.4). The buffer contained 50% (w/w) sucrose to reduce light scattering and improve the macroscopic orientation [37]. Reduced LD (LD^r^), which is a concentration and pathlength-independent quantity, was obtained by dividing the LD with the isotropic absorption (A_iso_). A_iso_ was recorded for each sample on a Varian Cary Bio 50 (Agilent Technologies, USA) spectrophotometer, between 185 nm and 600 nm, directly after the flow LD measurement.

### Construction of expression plasmids

Chemically synthesized oligonucleotides used in the cloning steps are listed in Additional file 1: Table S1. Human Oct4-PTD (wild-type) and mutants thereof (R16A and K13A/R16A), were cloned into a 6His-Cre fusion protein expressing construct [38], in two steps. First, sense and antisense oligonucleotides were annealed in fusion polymerase buffer (1X) (New England Biolabs, Inc.) by heating to 95°C for 5 minutes and cooling at room temperature for 15 minutes to generate a double-stranded (ds) oligo. Second, the ds-oligo was added to the N-terminus of the Cre fusion protein expression construct as NcoI-PstI fragment to generate Oct4-PTD-His-Cre wild type and mutant expression constructs.

### Purification of fusion proteins and translocation assays

Protein expression and purification of Oct4 protein and fusions thereof was carried out as previously described [28, 39, 40]. pSESAME-Oct4 vectors enable bacterial expression of histidine-tagged Oct4 fusion proteins carrying the CPP derived from TAT. For expression, BL21 (DE3) competent bacteria were transformed with pSESAME-Oct4 plasmids and protein production was induced by addition of Isopropyl-b-D-thiogalactopyranosid (IPTG; Life technologies, Carlsbad, CA). The bacteria containing recombinant protein were collected by centrifugation and lysed using disruption buffer (100 mM Tris, 1 mM EDTA, pH 8.0, 3 mM MgCl2) supplemented with 1 mg/ml lysozyme (Fluka Analytical, St. Gallen, Switzerland) as well as 10 U/μl benzonase (Merck, Darmstadt, Germany). The crude lysate was centrifuged and the pellets containing the inclusion bodies were washed repeatedly with washing buffer (50 mM Tris, 0.1 M NaCl, 0.5% Triton-X-100 (Sigma, Saint Louis, MO), 0.1% sodium azid, pH 8.0 and 0.1 M Tris, 2 mM EDTA, pH 8.0). The inclusion body fraction was solubilized using 8 M Urea, 50 mM Tris, 1 mM EDTA, 100 mM DTT and dialysed against a buffer comprising 6 M GuaHCL (pH 4.5). In a next step, the denatured protein was refolded by rapid dilution in 50 mM Na2HPO4, 1 mM EDTA, 5 mM GSG, 3 M Urea, 20% Glycerol, 1 M L-Arginine and 5% sucrose. The refolded protein was incubated with Ni-NTA agarose beads (Qiagen, Hilden, Germany) and concentrated by affinity chromatography. The protein was eluted with 50 mM Na2HPO4, 5 mM Tris, 500 mM NaCl, 250 mM imidazole (pH 7.8). The purity of the Oct4 protein was routinely controlled by SDS-PAGE (Additional file 2: Figure S2).

For the Oct4 translocation assay recombinant human Oct4 protein was dialyzed against protein transduction media (DMEM/F12, 2% FCS, 7,5% Serum Replacement, 100 mM b-mercaptoethanol, 1× NEAA, 1× Insulin-Transferrin-Selenium-Ethanolamine and 0.5% AlbuMAX. All media and cell culture supplements were purchased from Life technologies, Carlsbad, CA). Cultured CVI-5B and BJ fibroblast cells were incubated with 100 nM recombinant Oct4 protein for 3 hours. After removal of the protein transduction media cells were washed with PBS three times. Thereafter a Heparin (Sigma H3149) wash (0.5 mg/ml in PBS) was performed twice to remove non-internalized protein bound to the surface. Subsequently, cells were fixed in 4% paraformaldehyde for 10 min, blocked with 1% BSA and 0.1% Triton X-100 in PBS for 1.5 h and incubated with primary (anti-Oct-3/4, Santa Cruz Biotechnology sc-5279, 1:100) for 1.5 h and secondary antibody (Alexa 488) for 1 h. Nuclei were visualized by DAPI staining. Microscopy was done on a Leica DM IL LED FLUO inverted microscope in 40× and 63× magnification.

Functional assessment of internalized recombinant Cre-fusion protein was performed using the CVI-5B Cre reporter cell line as previously described [38].

## Results

### Oct4 contains a penetratin like CPP-domain

**Figure 1:**
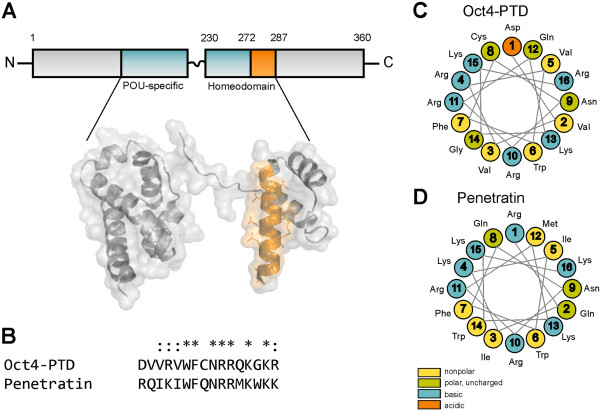
**Overview of the investigated peptides. (A)** Oct4-PTD derives from the third helix of the homeodomain (orange) of the transcription factor Oct4 (POU5F1), which possesses a POU-specific and a homeodomain [42], pdb: 3L1P. **(B)** The sequence alignment of Oct4-PTD and Penetratin shows great conservation, almost half of the residues are identical (*), more than two thirds of the peptide share at least same charge (* or :) **(C, D)** Helical wheel projection of Oct4-PTD or penetratin.

The cell penetrating peptide penetratin is derived from a DNA binding domain of a homeobox transcription factor. Oct4 belongs to the same class of transcription factors and also contains a bi-partite DNA-binding domain, the POU-specific domain (POUs), which recognises the octamer motif as well as the homeodomain (POUhd) (Figure 1A). Therefore, we aligned Oct4 and penetratin sequences and found a high degree of homology in the third helix of the respective homeodomains. The homology between the 16 aa of penetratin and the respective region of Oct4 is 7 out of 16 (43%) identities and 11 of 16 (68%) similarities (Figure 1B). Due to this similarity we termed this putative CPP Oct4-protein transduction domain (Oct4-PTD). The Oct4-PTD differs from penetratin mainly by having less lysines but more arginines – a feature that might be of advantage as far as uptake is concerned [41]. These differences are visualized by a helical wheel projection showing the differing distribution of the charges over the peptide suggesting diverging structural features of Oct4-PTD compared to Penetratin. Still, we considered the similarities to be sufficiently high to test Oct4-PTD for a putative activity as CPP.

### Oct4-PTD is taken up more efficiently than Penetratin by CHO-K1 cells

**Figure 2:**
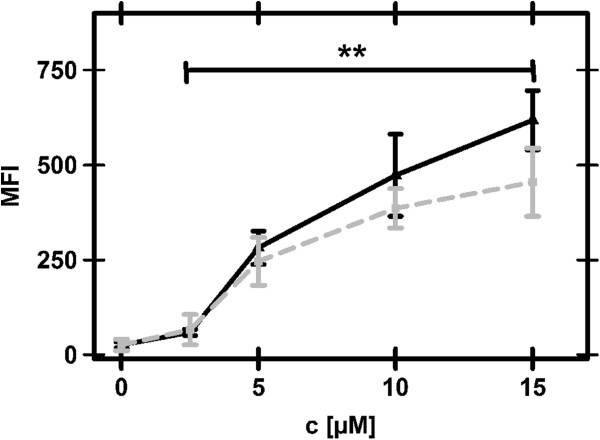
**Cellular uptake determined by flow cytometry.** Cells were incubated with 0, 2.5, 5, 10, 15 μM of FITC-labelled Oct4-PTD (black, solid) or Penetratin (gray, dashed) for 60 min at 37°C, washed with a heparin containing buffer to remove extracellular peptide and analysed for uptake. The results represent the mean fluorescence intensity (MFI) of 3 (15 μM) or 4 (2.5, 5, 10 μM) independent experiments (N = 3-4) with 4 biological replicates per experiment (n = 4). Compared to the untreated cells, both peptides show significant uptake even at the lowest concentration. **p-value ≤ 0.001.

In order to quantitatively compare uptake of Oct4-PTD to the well studied penetratin, we performed flow cytometry on N-terminally FITC labelled versions of the peptides. Uptake was analysed after 60 min incubation of CHO-K1 cells with different concentrations of peptides at 37°C and stringent washing with Heparin to remove externally bound peptide as described previously [31]. The cells efficiently took up both peptides in a concentration dependent manner (Figure 2). No saturation was observed in the applied concentration range, which varied from 2.5 μM to 15 μM. Relative quantification showed that Oct4-PTD displayed higher uptake efficiency than penetratin. This is visualised by subtracting the fluorescence values of penetratin from the ones of Oct4-PTD. Thus, in the 60 min exposure that we used, linearly increasing amounts of Oct4-PTD over penetratin were observed, reaching significance at 15 μM (Figure 2). Peptide concentrations below 2.5 μM did not result in detectable uptake signals (data not shown), possibly a result of the detection limit of our instrumentation.

### Uptake pattern and cellular distribution of Oct4-PTD

**Figure 3:**
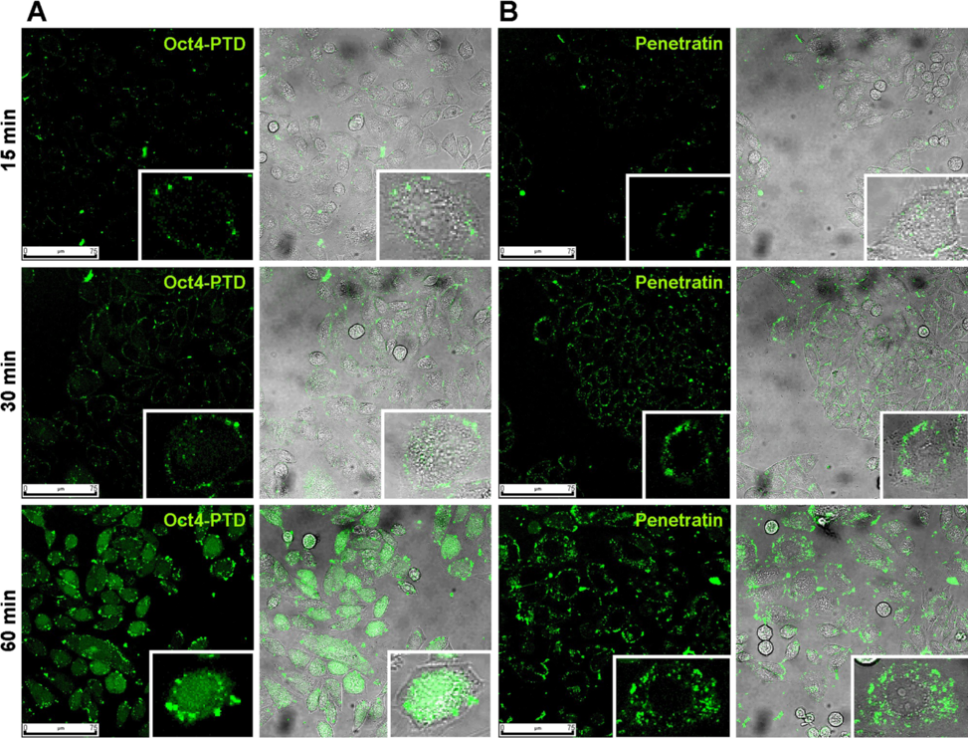
**Time-dependent uptake and differences in localization pattern.** Confocal images of live CHO-K1 cells incubated with **(A)** Oct4-PTD or **(B)** Penetratin for 15, 30 or 60 min at 37°C. Peptides were applied at a final concentration of 5 μM in serum free medium. Laser and photomultiplier settings were kept constant during the entire experiment; intensities are therefore comparable.

In order to visualize the cellular distribution of the labelled peptides, confocal live cell imaging was performed. After 15 min of cellular exposure to the CPPs, weak binding of both peptides to the plasma membrane was observed. At this time point no peptide seemed to be internalized, and in both cases a punctuate pattern at the membrane could be observed (Figure 3A). After 30 min a strong increase in fluorescence signal was seen (Figure 3B). Both peptides were not only bound to the membrane but had entered the cells. Furthermore, punctuate staining of the cytosol was observed for penetratin. In contrast, the Oct4-PTD signal, even though in part punctuate, appeared to be more diffuse in the cytoplasm and, surprisingly, also to be present in the nuclei. After 60 min of incubation, this difference in localization was even more pronounced. While most penetratin remained trapped in punctuate patterns excluded from the nuclei, possibly representing endosomes, Oct4-PTD was found throughout the cell (Figure 3).

### Oct4-PTD does not show cytotoxic effects

Since the applicability of CPPs is dependent on low or preferably absent toxic effects, we assessed cytotoxicity of the peptides using two different approaches. In the first approach, a telomerase-immortalized human adherent renal cell line, that still retains all characteristics of normal cells and has been widely used as a toxicity model system [30, 43], was exposed to the unlabelled peptides. No effect on cell viability and proliferation was detected with MTT for the tested peptides even at the highest concentration applied (Additional file 3: Figure S3). The second assay tested the hemolytic activity of both peptides on human red blood cells. Here, both peptides were applied either in their unlabelled or FTIC-labelled form. Again, no cytotoxic influence of the unlabelled peptides could be observed (Table 1). However, FITC-labelled penetratin and labelled Oct4-PTD showed around 7% and around 2% hemolysis respectively at the highest concentration (45 μM).

**Table 1:** **Hemolytic activity of FITC-labelled and unlabelled peptides**

Peptide	Concentration [μM]	% hemolysis
Oct4-PTD unlabelled	5	0
	15	0
	45	0.1
Oct-4PTD + FITC	5	0
	15	0
	45	2.0
Penetratin unlabelled	5	0
	15	0
	45	0
Penetratin + FITC	5	2.5
	15	5.0
	45	7.2

### Oct4-PTD is at least partly internalized via endocytosis

**Figure 4:**
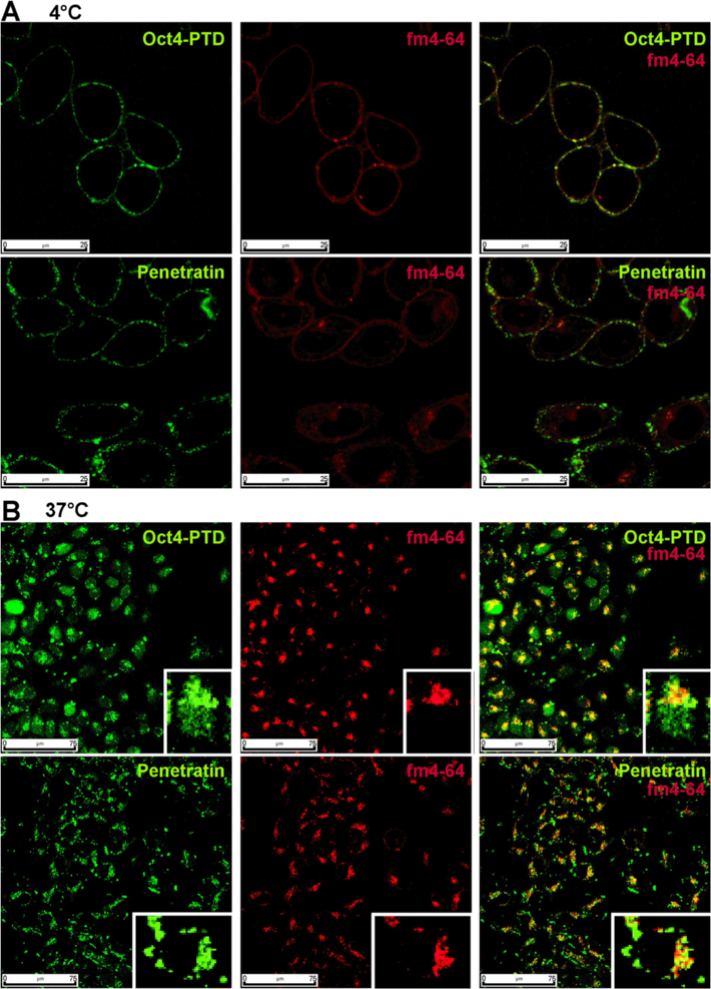
**Internalization is temperature-dependent and uses endocytic routes.** Confocal images of live CHO-K1 cells after incubation in the presence of the general endocytosis marker FM4-64 (red channel). Cells were incubated at **(A)** 4°C with 10 μM peptides or **(B)** 37°C using 5 μM (green channel), concentration of FM4-64 was 5 μg/ml in both cases. Laser and photomultiplier settings were kept constant for each temperature; intensities within the respective panels are therefore comparable.

Due to the striking differences in localization, we tested whether the uptake is at least in part mediated by direct translocation into the cells or if it rather relies on endocytic pathways. Therefore, we co-incubated the cells with peptide solutions and the general endocytosis marker FM 4-64 at 4°C or 37°C for 60 min and monitored the respective uptake behaviour using confocal microscopy. Since endocytosis is blocked at 4°C, no internalization of newly formed endosomes should be observed. Figure 4A shows that after 60 min of blocked endocytosis, neither Oct4-PTD nor penetratin was internalized. The peptides remained stuck at the plasma membrane and co-localized with extracellularly bound FM 4-64 marker. Thus, we did not detect any direct translocation of either peptide into the cells. At 37°C a clear partial co-localization of both FM 4-64 and both peptides can be seen (Figure 4B) indicating, at least in part, endocytic transport of the peptides into the cells.

### The alpha helical structure of Oct4-PTD is dependent on FITC

**Figure 5:**
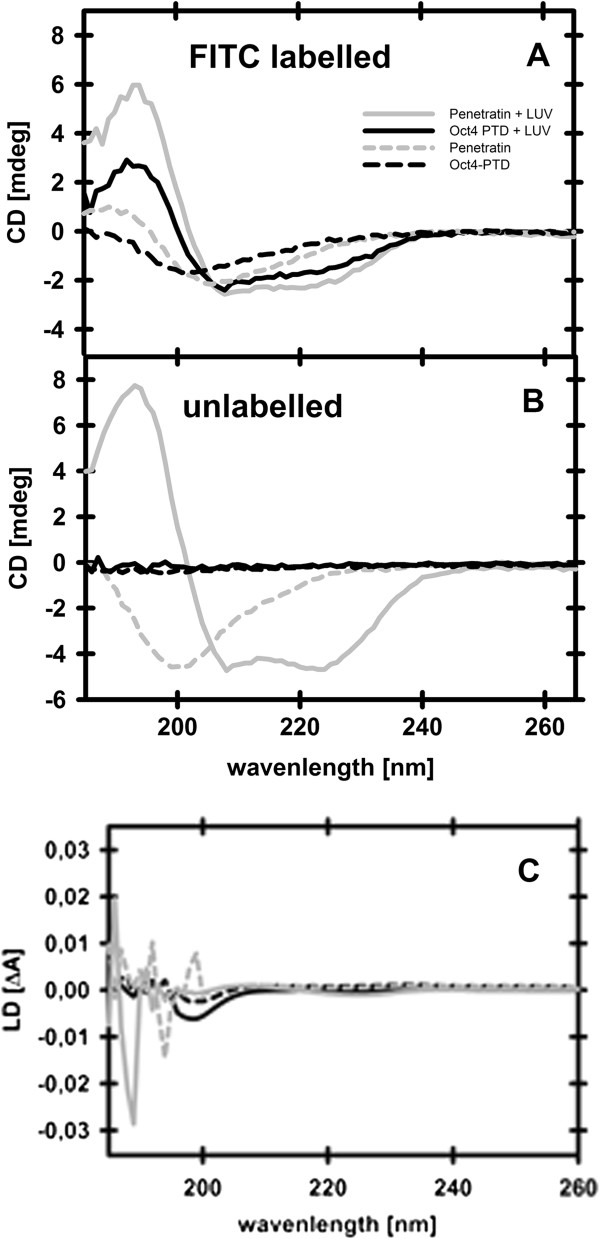
**Conformational changes upon binding to LUVs.** Circular dichroism spectra of 4 μM **(A)** FITC-labelled and **(B)** unlabelled Oct4-PTD (gray) and penetratin (black). Solid lines represent peptides in the presence of 0.4 mM POPG/POPC (80/20) LUVs, dotted lines show free peptide in solution. Measurements were performed in 10 mM phosphate buffer, pH 7.4. **(C)** LD spectra of penetratin (gray) and Oct4-PTD (black). Unlabelled peptides are represented as solid lines, FITC-labelled peptides as dashed lines.

CPPs can fold into distinct secondary structures, including α-helixes and random coils, upon interaction with lipid membranes. The resulting structures interact more or less strongly with the plasma membrane, leading to differences in uptake efficiency and cellular distribution [14]. Therefore, circular dichroism (CD) was measured to evaluate secondary structures of penetratin and Oct4-PTD, with and without fluorophore, in buffer and when bound to lipid vesicles. Both FITC-labelled peptides show a random coil structure in buffer. When bound to lipid vesicles, FITC-labelled penetratin and ditto Oct4-PTD show an evident α-helical signal, with a positive peak at 195 nm, and two negative peaks at 208 nm and 223 nm respectively (Figure 5A). If instead looking at the unlabelled peptides (Figure 5B), penetratin adopts an even more pronounced α-helix when bound to liposomes and an evident random coil when free in solution. However, the unlabeled Oct4-PTD shows an extremely weak, seemingly absent CD signal, indicating an unordered structure. The measurement was repeated with a three times higher peptide concentration, still rendering a very weak signal. The results show that a fluorescent label may induce structural changes in peptides, affecting the secondary structure of the peptide, both when free in solution or bound to lipid membranes.

### Peptide orientation upon binding to lipid model membranes

The peptide backbone absorption in the far-UV can be used to estimate the orientation of the peptide and its moieties compared to the lipid membrane. As seen in Figure 5C the LD signal for unlabeled penetratin is much more pronounced than for unlabeled Oct4-PTD, both regarding the tryptophan- and the α-helical transition moments. In an α-helix, the π-π* transition moments of neighbouring peptide bonds will split into two transition moments, rendering one parallel to the helix (200-210 nm) and one perpendicular to the helix (<200 nm). The indole ring of tryptophan has three transition moments, Bb at around 225 nm, La at around 270 nm and Lb at around 290 nm [44]. The positive peek at around 208 nm for penetratin points at this peptide being an α-helix, situated parallel to the membrane. At the same wavelength Oct4-PTD shows a signal very close to zero, meaning that this peptide does not form an α-helical structure, in accordance with the CD-measurement of unlabelled Oct4-PTD (see Figure 5B). Also the tryptophan transition moments point to this result. For Oct4-PTD the signal is overall very weak. Penetratin shows peaks for Bb at 225 nm and for the overlapping peaks of La and Lb between 230 and 300 nm, indicating that the secondary structure of penetratin has a higher degree of order compared to Oct4-PTD. The weak signal of Oct4-PTD also makes it difficult to evaluate its relative orientation compared with the membrane. The concentration and path length-independent reduced LD (LD^r^) is presented in Supplementary Information (Additional File 4: Figure S1).

### Oct4-PTD drives translocation of fused proteins

**Figure 6:**
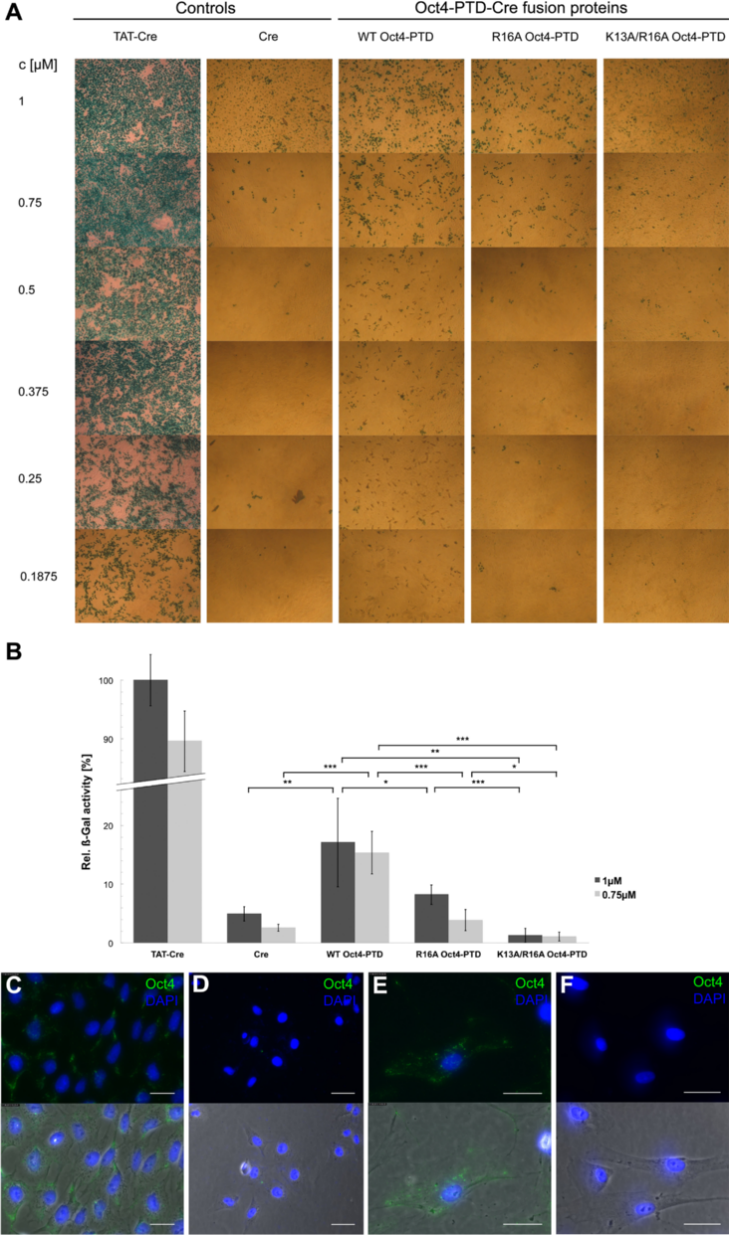
**Oct4-PTD is able to mediate uptake of protein cargo. (A)** Oct4-PTD-Cre fusion proteins are taken up by CV1-5B Cre reporter cells as determined by microscopy of cells stained positive for b-Gal activity. Increasing protein concentrations of recombinant wild type Oct4-PTD-Cre fusion protein (WT) and mutated versions of the Oct4-PTD (R16A as wells as K13A/R16A) in fusion with Cre, respectively, were subjected to a Cre recombinase assay where the intracellular activity of transduced protein is assessed by b-galactosidase activity [45]. TAT-modified Cre protein [47] and unmodified Cre protein carrying no transduction peptide served as controls (first two rows). Quantification of Cre-reporter assay outlined in **(B)** (n = 5). All values relative to TAT-Cre 1 μM. Error bars and n represent standard deviations and image sections quantified for b-Gal activity, respectively. Two-tailed t-test was used for statistical analysis. *p <0.05, **p <0.01, ***p < 0.001. **(C-F)** Detection of recombinant human Oct4 protein in mammalian cells after extracellular exposure. CV1-5B **(C)** and human BJ foreskin fibroblasts **(E)** were incubated with 100 nM recombinant human Oct4 protein for 3 hours, washed and subsequently stained for Oct4 and DAPI. **(D, F)** Untreated CV1-5B cells **(D)** and BJ fibroblasts **(F)** stained with first (anti-Oct3/4) and secondary antibody (Alexa 488) served as controls. Scale bar: 25 μm.

In order to test if Oct4-PTD is able to translocate other cargo than fluorescent labels into a cell and determine their intracellular activity after transduction, we used a Cre/loxP reporter system [45]. We generated a recombinant Oct4-PTD-Cre fusion protein as well as mutations in the Oct4-PTD as controls. The design of the mutants was based on a structure-activity relationship study of penetratin that led us to perform a R16A and a K13A/R16A exchange [46]. TAT-Cre, comprising the highly potent TAT transduction peptide in fusion with Cre served as a positive control [47]. After application of purified fusion proteins to CVI-5B reporter cells we observed a dose dependent activation of the loxP-modified reporter gene indicating Cre uptake via the Oct4-PTD (Figure 6A, B). Quantification of b-gal activity showed that the two Oct4-PTD mutatnts inverstigated did not result in b-gal activity above untagged Cre as observed by light microscopy (Figure 6B). However, our Oct4-PTD-Cre constructs were less efficient than the TAT-tagged Cre control.

### Oct4 translocates without the need of a cationic fusion tag

To test the ability of full length human Oct4 to enter mammalian cells, we generated recombinant human Oct4 without additional transduction tag. Recombinant Oct4 was administered to CVI-5B cells (Figure 6C) described above but also to human BJ foreskin fibroblasts (Figure 6E). After 3 hours incubation and a subsequent heparin wash to remove membrane bound protein, Oct4 protein was detected by immunostaining in both cell types (Figure 6C, E). Oct4 was particularly located in punctuate, perinuclear vesicle-like structures. This characteristic pattern has been widely described for transduced proteins and indicates efficient cellular uptake [48, 49]. Figure 6D, F show the respective negative controls, which were not exposed to recombinant Oct4 protein.

## Discussion

The present study reports the identification and characterization of a novel CPP derived from the homeodomain protein Oct4. The corresponding peptide Oct4-PTD, was found to be more efficiently taken up by cells than the well known CPP penetratin. In addition, not only uptake efficiency, but also intracellular localization varies between Oct4-PTD and penetratin. After 15 min of incubation, both peptides were bound to the plasma membrane in vesicular structures but did not enter the cells. 15 minutes later, the vesicular structures were for both peptides efficiently internalized. After 60 min, Oct4-PTD clearly showed diffuse staining within the whole cytoplasm as well as the nucleus, while penetratin still localized exclusively to cytoplasmic vesicles, excluded from the nucleus. The observed differences in uptake and intracellular localization might be associated with the higher number of arginine residues in Oct4-PTD. Even though Oct4-PTD has one cationic charge less than penetratin due to its N-terminal aspartate, the arginine-rich C-terminal part of the peptide still seems to have positive impact on the uptake as previously reported for penetratin [46]. Especially, the guanidinium head group of arginine has been shown to confer more efficient uptake of arginine, possibly caused by formation of bidentite hydrogen bonds with oxo-anions from e.g. sulfated sugars present in the extracellular matrix [50, 51]. Predominant cyotosolic and nuclear staining has been observed before with other CPPs or CPP-containing proteins in different cell lines [52–54]. Also, for lactoferrin-derived CPPs conjugated to Biotin-Streptavidin, nuclear localization in live cells was dependent on concentration of the peptide-cargo complex as well as the origin, since only peptide derived from human but not rat lactoferrin was detected in the nuclei [52]. Further, PDX-1, a pancreatic homeobox factor has been shown to be taken up by different target cell types and be transported to the nucleus (Koya 2008, Noguchi 2005). Therefore, testing the internalization ability and functional activity of the whole Oct4 protein in various cell types was a consequent step.

**Figure 7:**
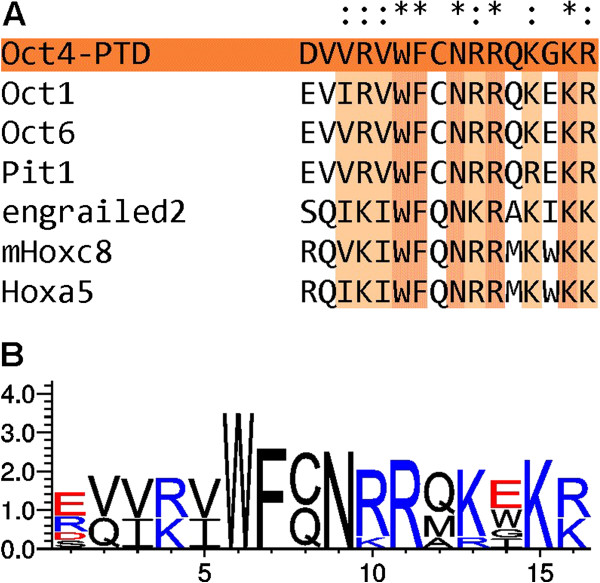
**Conservation of the 3rd helix of selected homeodomain transcription factors.** Comparison of the 3^rd^ helix of the homeodomain three transcription factors with known crystal structures (Oct1, Oct6 and Pit1) as well as three previously published CPPs. **(A)** Sequence alignments have been performed using T-coffee [55, 56], **(B)** weblogo illustrations were generated using weblogo 3.3 [57].

As the homology of homeodomains is evolutionary conserved, inherent protein transduction domains might well be a more general phenomenon for these transcription factors. Therefore, we have performed further sequence analysis and alignments (Figure 7) for the 3^rd^ helix of the homeodomain of other members of the POU family of transcription factors (Oct1, Oct6 and Pit1). We also included the aforementioned previously described homeodomain-derived CPPs (Engrailed2, Hoxa5, Hoxc8). As expected, Figure 7A shows that these sequences are highly homologous. Still, there are some considerable differences in the C-terminal region of the peptides. Especially at position 14 the variation between charges and hydrophilic nature of the amino acid residues are evident (Figure 7B). This makes it difficult to predict a potential uptake of other homeodomain proteins.

How might the more efficient uptake and distribution into cytoplasm and even nucleus be explained when looking at the sequence and the secondary structure of the peptides? When bound to lipid membranes, both labelled Oct4-PTD and penetratin adopt α-helical conformations, and for the unlabelled peptide the signal is evidently more pronounced than for penetratin. This difference becomes even more evident with the LD measurements, showing both higher degree of orientation and a more prominent α-helical signal for penetratin. One reason for the higher orientation monitored for penetratin could be that this peptide has two tryptophans, whereas Oct4-PTD has one. Also, the position of tryptophan and other hydrophobic, bulky residues could have impact. When comparing the LD results with the helical projection (Figure 1C), it is seen that there is an accumulation of hydrophobic residues on one side of penetratin, compared with the more dispersed pattern for Oct4-PTD, which could result in higher degree of orientation. Even if it still remains unclear what the role of a more ordered structure in regard to uptake would be, it is clear that the two peptides interact differently with membranes, which might affect the uptake into cells. The relative importance of the secondary structure for uptake is to date not fully understood [58]. The lactoferrin derived CPP uptake seems to strictly depend upon an α-helical conformation [52] whereas Tat enters in a coiled conformation [58]. Different helix-disruptive mutations of penetratin showed similar uptake efficiencies as the wild type peptide [59] while the more helical PenArg peptide had less efficient uptake [60]. It is therefore very likely that the less ordered unlabelled Oct4-PTD is internalized and may be a good vector for cargo molecules, although, we do at the moment not know whether the FITC induced structural difference will have impact on the uptake of Oct4-PTD. In any case, it was surprising to see such a large impact on the conformation of the Oct4-PTD by the FITC label, and that for any experiment performed on CPPs, structural analysis by CD may be an important tool to guarantee comparability of data from studies using labelled and unlabelled peptides for different types of analyses.

To understand if Oct4-PTD is directly translocated through the membrane or if it is taken up by an endocytic mechanism, co-localization studies with the general endocytosis marker FM4-64 were performed at 4°C and 37°C. When endocytosis was blocked (4°C), no uptake of either peptide was observed suggesting no direct translocation mechanism. For penetratin these data support studies that have reported temperature-dependent uptake using a similar setup [61, 62] but also stand in conflict with other studies that used either fixed cells or a mass spectrometry based approach [10, 11]. Still, formation of vesicles on the outer side of the cell membrane and in part co-localization with FM4-64 was observed even at low temperature. When incubating the cells at 37°C co-localization of both peptides with FM4-64 was observed. FM4-64 has strong affinity towards the plasma membrane and stains any vesicle that will bud off from it [63]. Consequently, it is not surprising that individual red staining can be seen in both cases. For penetratin it has been shown that its uptake is clathrin-independent and mainly uses the macropinocytotic route [41, 64]. Similarly, Oct4-PTD may also use distinct endocytotic routes for cellular entry, which remain to be explored. The nuclear and diffuse cytoplasmic staining also would suggest that FITC-labelled Oct4-PTD is not only an efficient new CPP but that it is also able to escape the endosomes, probably by destabilizing the vesicular membrane in a similar fashion as the endosomolytic EB1 peptide [65]. These results lead us to propose the following model of mechanism of uptake of Oct4-PTD: initial binding to the membrane and vesicle formation leads to internalization, mainly driven by endocytosis. After around 30 min, many vesicles have already entered the cell and Oct4-PTD starts to distribute into the cytosol and eventually escapes the endosomes.

Further confirmation that the Oct4-PTD exerts cellular translocation activity is provided by a Cre/loxP-based reporter system that has been widely used to study protein transduction [31, 38]. This assay showed weak yet clear b-Gal staining of reporter cells treated with the wild type Oct4-PTD-Cre-fusion protein whereas untreated cells remained unstained. Notably, the Cre-activity of the Oct4-PTD-fused protein was less pronounced than for the TAT-PTD. This result indicates that the translocation activity of the Oct4-PTD is weaker than that of TAT-PTD at least in CV1-5B cells.

Finally, we demonstrate that full length human Oct4 was found to enter cells without additional fusion tag and remained predominantly stuck in vesicles. This observation correlates well with previously published data reporting that the majority of the transduced protein can be detected in perinuclear vesicular structures [48, 49]. From there a subfraction of internalized protein is able to escape via an endosomal release route that is not fully understood at molecular level.

In conclusion, we propose that Oct4-PTD might be a valuable candidate for further investigations concerning its ability to transport different therapeutic cargo molecules such as siRNAs intracellular proteins into cells. Moreover, our results also suggest that the human Oct4 protein might be able to actively enter cells via the Oct4-PTD, further supporting the concept of ‘signalling homeo-proteins’ found in mammalian but also plant cells [66, 67]. Finally, these data support the idea that the addition of a cationic fusion tag to recombinant Oct4 currently applied for iPS reprogramming can be avoided. This might contribute to produce integration free induced pluripotent stem cells [25, 27, 39].

## Electronic supplementary material

Additional file 1: Table S1: Cre-fusion protein expression vector - oligo design. (DOC 36 KB)

Additional file 2: Figure S2: Purification of Oct4-PTD-Cre fusions. The estimated size of the given constructs was 42.6 kDa. Annotation of the nomenclature: L1A: R16A; L2A: K13A/R16A; L4A: wild type Oct4-PTD. (PDF 455 KB)

Additional file 3: Figure S3: Cellular proliferation capacity is not altered by peptides. MTT assay of RPTEC/TERT1 cells after 1 or 24 hours of incubation with the respective peptides. The bars represent the endpoints of each measurement at a concentration of 80 μM. 1-way ANOVA was calculated using Friedmann Test in GraphPad Prism. (PNG 73 KB)

Additional file 4: Figure S1: LDr of labelled and unlabelled peptides. Reduced linear dichroism spectroscopy in the presence of LUVs. Black lines represent Oct4-PTD, gray lines penetratin either unlabelled (solid) or labelled (dashed). (TIFF 647 KB)
