# Supplementary material for: Characterization of a novel cell penetrating peptide derived from human Oct4
Source: Cell Regen. 2014 Jan 31;3:2. doi: 10.1186/2045-9769-3-2 (PMC4230757; doi:10.1186/2045-9769-3-2)
Supplement: Supplementary file 1 — Additional file 1: Table S1: Cre-fusion protein expression vector - oligo design. (DOC 36 KB) [file 13619_2013_17_MOESM1_ESM.doc]

# Supplementary Information

Additional file 1: **Table S1. Cre-fusion protein expression vector - oligo design**

| **Clone name** |  | **Oligonucleotide sequence** |
| --- | --- | --- |
| Oct4-PTD(wild-type) His | Sense | **5'** *CCATGG*gc**gatgtggtccgagtgtggttctgtaaccggcgccagaagggcaagcga**CATCACCATCACCATCAcggcatgggcg*CTGCAG* **3'** |
|  | Antisense | **5'** *CTGCAG*cgcccatgccgTGATGGTGATGGTGATG**tcgcttgcccttctggcgccggttacagaaccacactcggaccacatc**gc*CCATGG* **3'** |
| Oct4-PTD(K13A) His | Sense | **5'** CCATGGgc**gatgtggtccgagtgtggttctgtaaccggcgccaggctggcaagcga**CATCACCATCACCATCAcggcatgggcgCTGCAG **3'** |
|  | Antisense | **5'** CTGCAGcgcccatgccgTGATGGTGATGGTGATG**tcgcttgccagcctggcgccggttacagaaccacactcggaccacatc**gcCCATGG **3'** |
| Oct4-PTD(R16A) His | Sense | **5'**CCATGGgc**gatgtggtccgagtgtggttctgtaaccggcgccagaagggcaaggcc**CATCACCATCACCATCAcggcatgggcgCTGCAG **3'** |
|  | Antisense | **5'**CTGCAGcgcccatgccgTGATGGTGATGGTGATG**ggccttgcccttctggcgccggttacagaaccacactcggaccacatc**gcCCATGG **3'** |
| Oct4-PTD(R16A & K13A) His | Sense | **5'**CCATGGgc**gatgtggtccgagtgtggttctgtaaccggcgccaggctggcaaggcc**CATCACCATCACCATCAcggcatgggcgCTGCAG **3'** |
|  | Antisense | **5'**CTGCAGcgcccatgccgTGATGGTGATGGTGATG**ggccttgccagcctggcgccggttacagaaccacactcggaccacatc**gcCCATGG **3'** |

**Annotations: Oct4 peptide:** bold and lowercase, **His tag:** Uppercase and underlined, **CCATGG (NcoI) and CTGCAG(PstI):** Restriction enzyme

# Supplementary figure Legends

**Figure S1 LD^r^ of labelled and unlabelled peptides**

Reduced linear dichroism spectroscopy in the presence of LUVs. Black lines represent Oct4-PTD, gray lines Penetratin either unlabelled (solid) or labelled (dashed).

**Figure S2 Purification of Oct4-PTD-Cre fusions**

The estimated size of the given constructs was 42.6 kDa. Annotation of the nomenclature: L1A: R16A; L2A: K13A/R16A; L4A: wild type Oct4-PTD

**Figure S3 Cellular proliferation capacity is not altered by peptides**

MTT assay of RPTEC/TERT1 cells after 1 or 24 hours of incubation with the respective peptides. The bars represent the endpoints of each measurement at a concentration of 80 µM. 1-way ANOVA was calculated using Friedmann Test in Prism graph.
