# Supplementary figures and images for: Characterization of a novel cell penetrating peptide derived from human Oct4
Source: Cell Regen. 2014 Jan 31;3:2. doi: 10.1186/2045-9769-3-2 (PMC4230757; doi:10.1186/2045-9769-3-2)

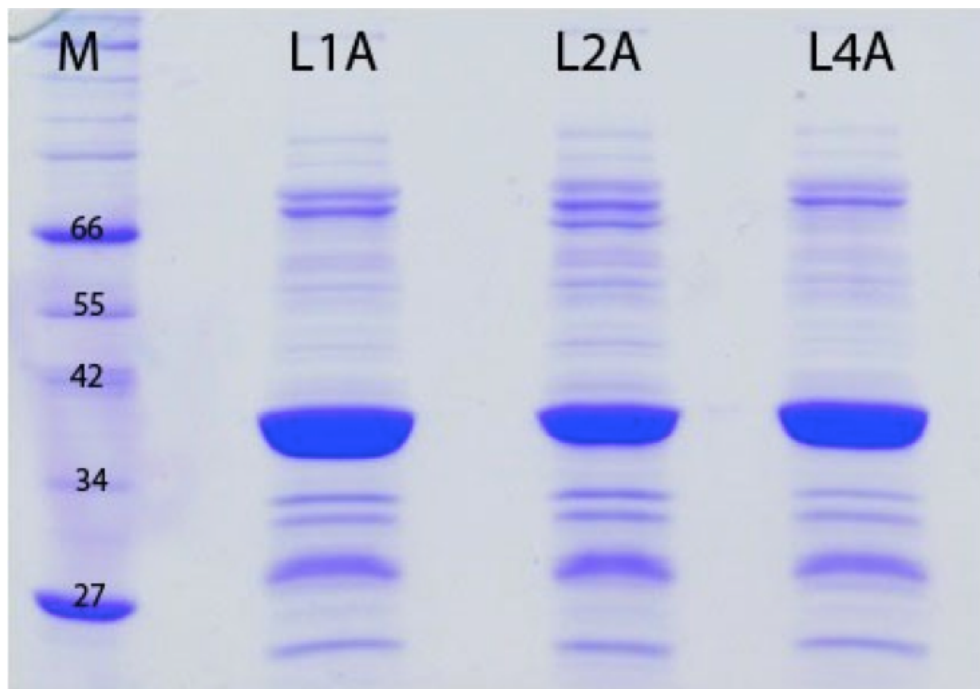

Supplement: Supplementary file 2 — Additional file 2: Figure S2: Purification of Oct4-PTD-Cre fusions. The estimated size of the given constructs was 42.6 kDa. Annotation of the nomenclature: L1A: R16A; L2A: K13A/R16A; L4A: wild type Oct4-PTD. (PDF 455 KB) [file 13619_2013_17_MOESM2_ESM.pdf]

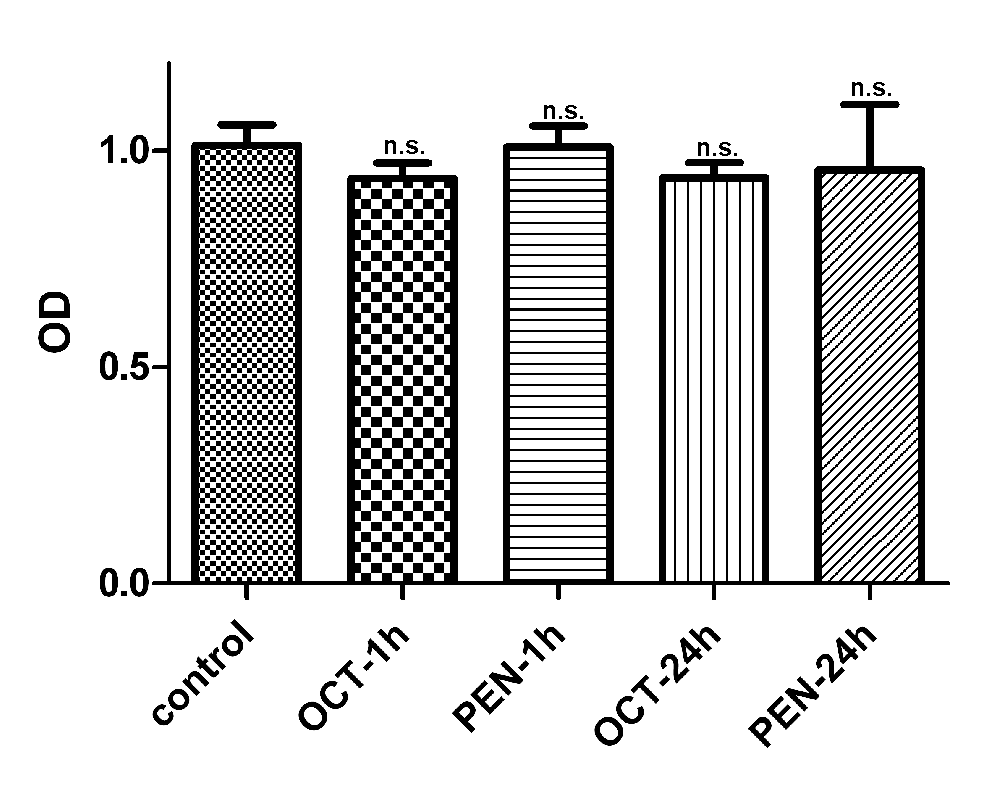

Supplement: Supplementary file 3 — Additional file 3: Figure S3: Cellular proliferation capacity is not altered by peptides. MTT assay of RPTEC/TERT1 cells after 1 or 24 hours of incubation with the respective peptides. The bars represent the endpoints of each measurement at a concentration of 80 μM. 1-way ANOVA was calculated using Friedmann Test in GraphPad Prism. (PNG 73 KB) [file 13619_2013_17_MOESM3_ESM.png]

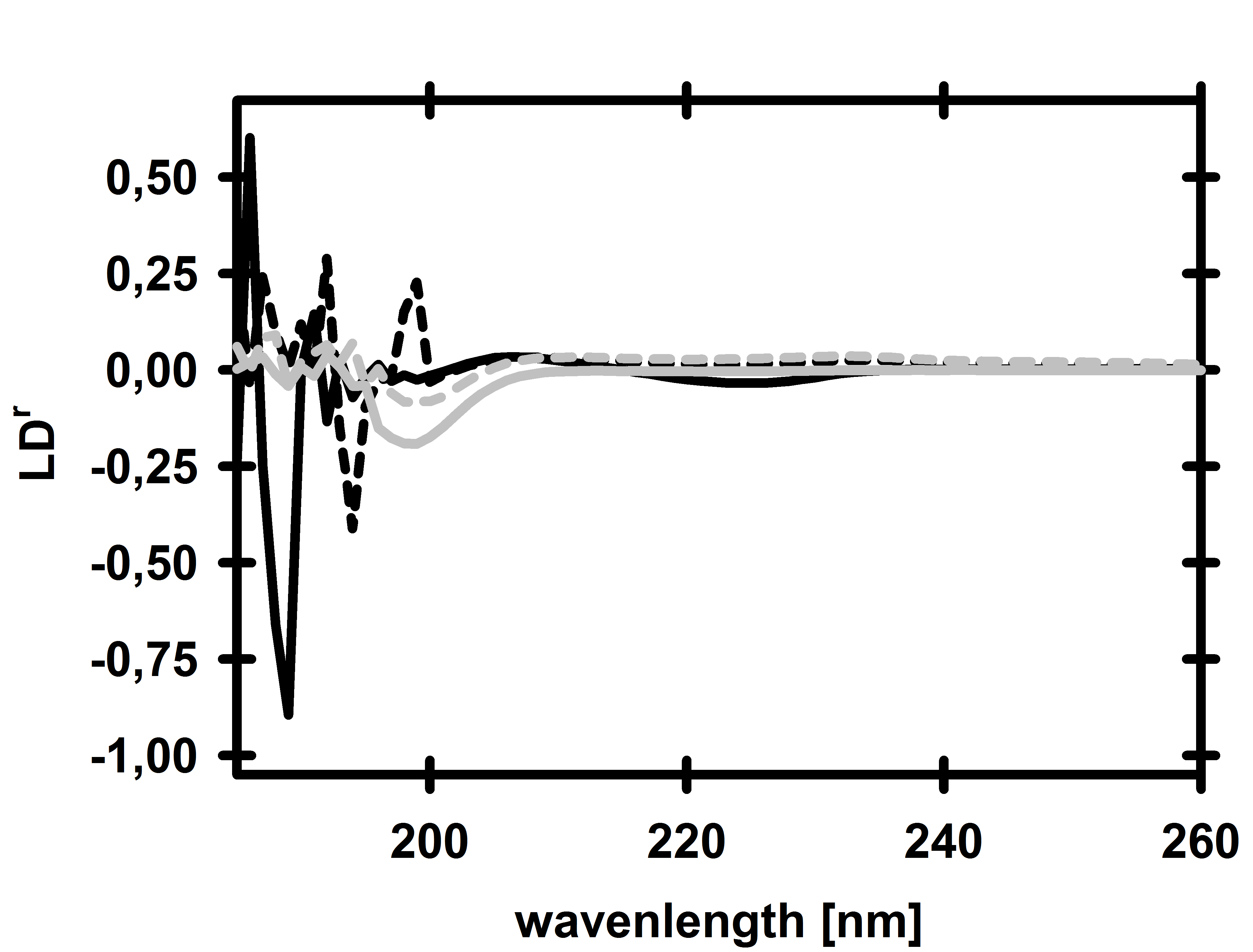

Supplement: Supplementary file 4 — Additional file 4: Figure S1: LDr of labelled and unlabelled peptides. Reduced linear dichroism spectroscopy in the presence of LUVs. Black lines represent Oct4-PTD, gray lines penetratin either unlabelled (solid) or labelled (dashed). (TIFF 647 KB) [file 13619_2013_17_MOESM4_ESM.tiff]
